# Perinatal depressive symptoms often start in the prenatal rather than postpartum period: results from a longitudinal study

**DOI:** 10.1007/s00737-020-01017-z

**Published:** 2020-02-04

**Authors:** Marsha Wilcox, Beth Ann McGee, Dawn F. Ionescu, Marie Leonte, Lauren LaCross, Jenna Reps, Kevin Wildenhaus

**Affiliations:** 1 Janssen Research & Development, Titusville, NJ USA; 2 BabyCenter, San Francisco, CA USA; 3 Janssen Research & Development, La Jolla, CA USA

**Keywords:** Perinatal depression, Longitudinal study, EPDS, Internet research

## Abstract

Depressive symptoms during and after pregnancy confer risks for adverse outcomes in both the mother and child. Postpartum depression is traditionally diagnosed after birth of the child. Perinatal depression is a serious, prevalent heterogeneous syndrome that can occur during the period from conception through several months after childbirth. Onset and course are not well understood. There is a paucity of longitudinal studies of the disorder that include the antenatal period in population-based samples. We used an Internet panel of pregnant women recruited in 2 cohorts; 858 ascertained in the first and 322 ascertained in the third trimesters of pregnancy. We recruited the second cohort in order to assure sufficient sample to examine depressive symptoms later in pregnancy and in the postpartum period. Assessments included standard psychometric measures, health history, and pregnancy experience. The Edinburgh Postnatal Depression Scale was used for the assessment of depressive symptoms. Nearly 10% of women entered the pregnancy with depressive symptoms. Prevalence was about the same at 4 weeks and 3 months postpartum. During pregnancy, prevalence increased to 16% in the third trimester. Among incident cases, 80% occurred during pregnancy, with 1/3 occurring in the first trimester. We describe predictors of incident depressive symptoms and covariates associated with time-to-onset which include health history (psychiatric and medical) and social support covariates. The majority of incident depressive symptoms occur during pregnancy rather than afterward. This finding underscores the mandate for mental health screening early in pregnancy and throughout gestation. It will be important to find safe and effective interventions that prevent, mitigate, or delay the onset of depressive symptoms that can be implemented during pregnancy.

## Introduction

According to The World Health Organization, maternal mental health constitutes a major public health challenge (Maternal and Child Mental Health n.d.). Postpartum depression (PPD), traditionally diagnosed after the birth of the child, occurs in 12–20% of all pregnancies (Maternal and Child Mental Health n.d.). In the USA in 2015, more than 750,000 women saw a physician or were hospitalized for PPD (National Center for Health Statistics. National Hospital Discharge Survey 2015; National Center for Health Statistics. National Ambulatory Medical Survey 2015).

PPD may be an inadequate descriptor of maternal depression because depression onset can happen in either the antenatal or postpartum periods. Recent research suggests that a significant portion of pregnancy-related depression manifests in the antenatal period (Wisner et al. 2013). Depression in the perinatal period can negatively shape the physiological and psychological health of both the mother and infant, leading to significant morbidity (Goodman et al. 2011; Grote et al. 2010). The term, “perinatal depression” (PND) is a more accurate and encompassing term. PND is a serious and prevalent condition (Stuart-Parrigon and Stuart 2014; O’Hara and Wisner 2014).

Symptoms of PND can mirror those of major depressive disorder (MDD) and may include the following: depressed mood, anhedonia, insomnia, poor appetite, impaired concentration, guilt, helplessness, tearfulness, somatic symptoms, and, in some cases, suicidal thoughts and/or self-harm. (Lee and Chung 2007). The 5th version of the *Diagnostic and Statistical Manual for Mental Illness* (DSM-5) (American Psychiatric Association 2013) requires endorsement of 5/9 symptoms of depression in the perinatal period to meet disease criteria.

However, two women who both meet criteria for PND may endorse very different symptoms, potentially indicative of underlying disease subtypes. Previous research has identified various subtypes of perinatal depression, characterized by distinct symptoms, severity, and onset (Drozd et al. 2018). The identification of subtypes will inform diagnosis, prognosis, and treatment of PND—ultimately improving the overall health of mothers and their children.

In a prior work re-analyzing an international cross-sectional dataset, we examined item response on the Edinburgh Postnatal Depression Scale (EPDS) and identified five distinct subtypes, marked by variation in mood, anxiety, and anhedonia. We also saw differences by time of onset (Putnam et al. 2017).

In a recent report, Stuart et al. (Stuart-Parrigon and Stuart 2014) cite several limitations in current literature including the use of cross-sectional studies and limiting the focus to a small number of risk factors. Population-based studies afford the ability to assess unmet medical need, not possible in clinic-based work. There are only a few population-based longitudinal studies to date.

The Avon Longitudinal Study of Pregnancy and Childhood (ALSPAC) used the EPDS at pregnancy weeks 18 and 32 and again at 8 and 32 weeks postpartum. The scores were reported as a binary outcome, depressed or not, using at cut-point of 12 (Fergusson et al. 1996). While this study had assessments during the pregnancy and in the postpartum period, the number of women entering the pregnancy with depressive symptoms or with onset in the first trimester was unknown.

The Etude du DÉveloppement des Nouveau-nés (EDEN) study followed French mothers and their offspring from two health centers from pregnancy week 24 through 12 months postpartum to examine determinants of child development and health. Depressive symptoms were assessed using the Center for Epidemiologic Studies–Depression scale (CES-D). Postnatal depression was assessed with the EPDS. They observed 22.5% with depressive symptoms during pregnancy and 13.6% with postnatal depressive symptoms (Melchior et al. 2012).

The aim of the Finnish study FinnBrain was to examine the effects of perinatal and early life stress on psychiatric and somatic illnesses. The sample was ascertained from three maternal welfare clinics in a single area. The prenatal stress (PS) indicator was a composite of mood (EPDS) and anxiety measures. The EPDS cut-point for depressive symptoms was 12 or higher. The prenatal stress case group was 20% of the sample. While this study had assessments in both the second and third trimesters, the number entering the pregnancy with depressive symptoms or with onset in the first trimester was unknown (Karlsson et al. 2018).

The Child-Sleep Cohort, also in Finland, was recruited in a single hospital district. Baseline assessments occurred at week 32 with follow-up occurred up to 2 years postpartum. The top decile of the CES-D was used to indicate depressive symptoms. The results of this study showed that depressiveness and insomnia are often comorbid (Paavonen et al. 2017).

A study in Singapore, Growing Up in Singapore Towards Healthy Outcomes (GUSTO), was a longitudinal parent-offspring cohort with extensive phenotyping of all participants. The primary objective was to evaluate influences on early development and metabolic compromise. The authors also examined maternal influences on the offspring including depression. Participants were recruited in the first trimester and followed for at least 3 years after birth. The depressive symptoms were assessed at 26 weeks; 7.2% scored 15 or higher and again at 3 months postpartum (10.4% with cut-point of 13+) (Soh et al. 2014; Chong et al. 2014).

Our goal was to address some of the limitations in existing literature with a repeated-measures longitudinal study including medical and psychiatric history, pregnancy experience, family and social support, mood, anxiety, sleep, and social support with assessments beginning early in pregnancy (weeks 4–10) and continuing through 3 months postpartum.

## Aim

The aim of the study was to describe perinatal depressive symptoms, including onset and trajectory.

## Methods

### Sampling frame

The sampling frame was an online panel recruited by BabyCenter, a Johnson & Johnson-owned website for pregnant women, new mothers, and young families (https://www.babycenter.com/). In the USA, 3 out of 4 new or expectant mothers online use BabyCenter each month.

### Sample

The US sample included pregnant women age 18+. Women on the BabyCenter site were invited to participate at random. In this convenience sample, participants were followed through pregnancy and 12 weeks into the postnatal period in 2 cohorts. The first was ascertained in the first trimester, the other in the third.

Of the 1179 participants, 858 were ascertained between weeks 4 and 10 and, 321 between weeks 28 and 33. Both cohorts were followed through 3 months postpartum. The study ran from August 2016 to September 2017. We had IRB approval for this work.

### Assessments

This study included 8 standard psychometric measures (Chorwe-Sungani and Chipps 2017):

1. The *Edinburgh Postnatal Depression Scale* (EPDS). The EPDS is a frequently used measure of postpartum mood (Putnam et al. 2017). Scores on the EPDS were not different from scores on the PHQ-9 in a study designed to evaluate the EPDS for assessing MDD sample of pregnant women (Flynn et al. 2011).
  The self-harm item was omitted because we could not provide clinical assistance for women endorsing suicidal thoughts. The 9-item scale was categorized as follows: “Unaffected” (0–7), “Low probability MDD” (8–11), “Possible MDD” (12–13), and “Probable MDD” (14 or higher). This categorization varies slightly from published studies because we used a 9- rather than 10-point scale.
  Incident MDD was defined as an EPDS score of 14+ with no prior score in that range. It was assessed at the end of each trimester, immediately following birth, and 3 months postpartum (Tables 1 and 2). High Probability MDD at baseline was considered prevalent MDD.
2. The *Generalized Anxiety Disorder-7 Item* (GAD-7) is a 7-item self-report instrument designed to assess anxiety during the previous 2-week period. It is useful for measuring change over time (Spitzer et al. 2006).
3. The *DSM-5 Anxious Distress Specifier* (ADS) for MDD includes 5 items, 4 of which are in the GAD-7. We added an item to assess feelings of loss of control for the ADS (American Psychiatric Association 2013).
4. The *State-Trait Anxiety Index* (STAI): we used a 6-item short form developed in a sample of pregnant women (Marteau and Bekker 1992).
5. The *Patient Reported Outcomes Measurement Information System* (PROMIS): we used the scales for emotional support (4 items), pain (4 items), emotional distress–anxiety (4 items), sleep disturbance (3 items), and sleep-related impairment (8 items) (Cella et al. 2007).
6. The *Perceived Stress Scale* (PSS): we used the 4-item abridged version (Cohen et al. 1983).
7. The *Obsessive-Compulsive Inventory–Revised* (OCI-R) is a commonly used 18-item self-report scale measuring symptoms in 6 subscales including washing, checking, neutralizing, obsessing, ordering, and hording (Abramowitz and Deacon 2006).
8. The *Patient Health Questionnaire-2 Item* (PHQ-2): this 2-item scale was designed to be used as a screen for major depression (Kroenke et al. 2003).
9. The *Perinatal PTSD–Modified* (PTSD), a 14-item scale assessing post-traumatic symptoms related to birth and the postnatal period (Callahan et al. 2006), was used.

**Table 1:** Assessment sets

		Core + baseline	PROMIS + PHQ2	Core − GAD-7	Core + OCI-R	Core + final
	Measurement set	1	2	3	4	5
EPDS	Edinburgh Postnatal Depression Scale^a^ (9)	✓		✓	✓	✓
PSS	Perceived Stress Scale (4)	✓		✓	✓	✓
STAI	Sate-Trait Anxiety Inventory (6)	✓		✓	✓	✓
GAD-7	Generalized Anxiety Disorder-7 (7)	✓		–	✓	✓
PROMIS scales	Emotional support (4)	✓		✓	✓	✓
	Pain interference (4)		✓			
	Sleep disturbance (4)		✓			
	Sleep-related impairment (8)		✓			
	Anxiety (4)		✓			
	Health history	✓				
	Demographic profile	✓				
PHQ-2	Patient Health Questionnaire-2 (2)		✓			
OCI-R	Obsessive-Compulsive Inventory–R (18)				✓	✓
PTSDM	Perinatal PTSD-Modified (14)					✓
	Birth experience					✓

^a^Self-harm item omitted

**Table 2:**
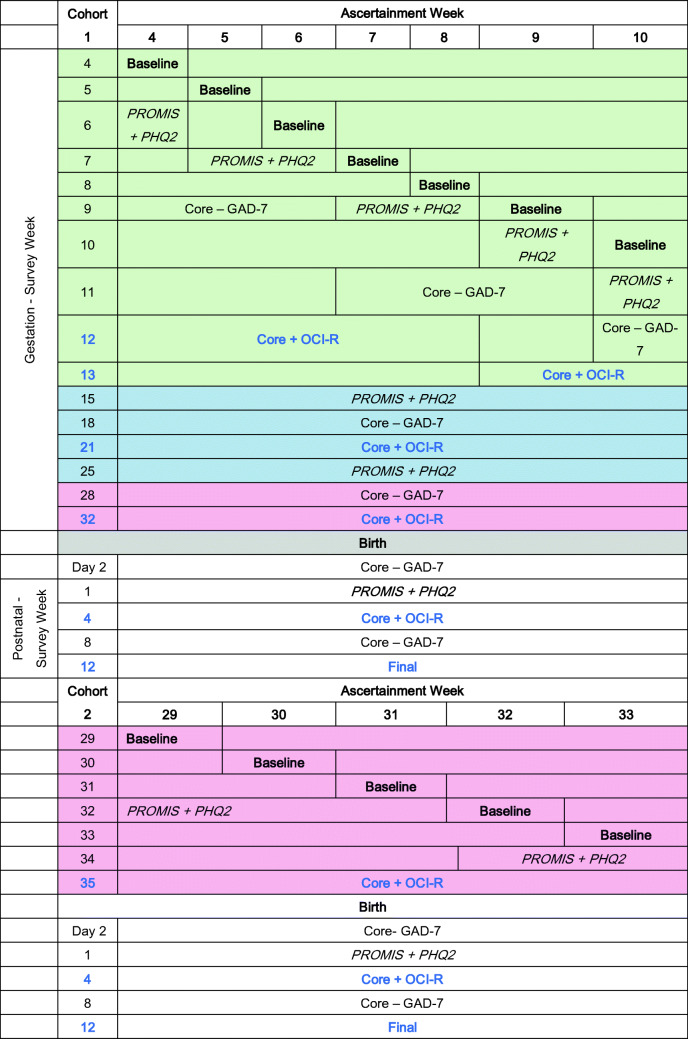
Ascertainment and assessment timing (T1: weeks 1–12, T2: 13–26, T3: 27–birth)

The assessments also included personal and family health history, pregnancy and birth experience and demographics (supplemental material).

### Assessment administration

Instruments were presented to respondents in 5 overlapping sets to reduce respondent burden. Participants in cohort 1 had the opportunity to respond at 10 timepoints before the birth of their children; cohort 2 had 3. Both cohorts were invited to complete 5 postpartum assessments. A list of assessments is in Table 1; timing of administration is in Table 2.

The core set of instruments included the EPDS, PSS, STAI, GAD-7, and the PROMIS Emotional Support scale. The Baseline assessment added personal and family health history as well as demographic information. The second set of assessments contained four of the PROMIS scales (Pain Interference, Sleep Disturbance, Sleep-Related Impairment, and Anxiety) and PHQ-2 to assess mood. The third set used the core measures without the GAD-7. The fifth and final assessment sets added the OCI-R to the core measures. The final measurement also added the PPTSD-M and an inventory about the birth experience and newborn health.

All responses were web-based. This allowed us to present assessments at the same week of pregnancy for each respondent. Women in cohort 1 were generally asked to complete the second assessment set using the PROMIS subscales and the PHQ-2 the week following entry into the study. One or 2 weeks later, the third set (core measures without the GAD-7) was presented. Most women in cohort 1 were near the end of their first trimester by this point, when core assessments were presented with the OCI-R (fourth set). The assessments in the second and third trimesters and postpartum periods occurred at the same week of pregnancy for each woman. Assessments were presented to women in cohort 2 in a similar manner.

Each assessment began with general questions about pregnancy experience and preparation for the birth of the child. Complete questionnaires can be found in the Supplemental Material.

### Incentives for participation

A points system was used to maintain engagement in the study. Points were awarded for each assessment completed in a system where 1 point = $1; more points were awarded for the longer assessments. After a participant accumulated 25 points, she could redeem them for a $25 gift card. Points held no additional value for participants. In addition to the point incentive system, participants earned entry into a sweepstakes for a $1000 gift card after every completed assessment. The drawing was conducted at the conclusion of the study and separately for each cohort.

At intervals during the study, summary information about the study was posted on the study website. A brief summary of results was posted afterward.

### Statistics

These results focus on describing perinatal depressive symptoms including onset and trajectory in cohort 1. Univariate, bivariate, and multivariate descriptive statistics were used. We used logistic regression to describe any incident depressive symptoms (binary) with a stepwise approach, entry criteria was *p* < 0.1. Proportional hazards regression was used to describe time-to-onset of depressive symptoms, with onset week as the measure of time. Both the fact of depressive symptoms (logistic regression) and time-to-onset (proportional hazards) were examined in the context of a set of covariates. Independent variables included (1) pregnancy experience at baseline (nausea, difficulty eating, difficulty managing weight, fatigue, back pain, gestational diabetes, headaches or migraines, trouble sleeping, mood swings, anxiety, preeclampsia, high blood pressure); (2) prior parity; (3) medical history (allergies diabetes (any), fertility problems, fibromyalgia, hypertension, irritable bowel syndrome, migraines, obesity sleep disorders, substance addiction, endometriosis, bipolar disorder, depressive symptoms, eating disorder, generalized anxiety disorder, obsessive compulsive disorder, panic attacks, postpartum depressive symptoms, post-traumatic stress disorder, other emotional/mental health disorders); and (4) past year experiences (death of a family member or close friend, ended a relationship, financial hardship, legal problem, miscarriage, moved, personally lost a job, partner lost a job, victim of a natural disaster, victim of a non-violent crime, victim of a violent crime). SAS version 9.4 was used for the analyses (SAS Software n.d.).

## Results

### Sample disposition

The survey invitation appeared on the site 476,863 times. It is impossible to know who saw it or how many times it was viewed. There were 5843 women who clicked on the survey invitation; 86% (5028) began the survey. Among those, 1557 (31%) qualified for the study. Table 3 shows the reasons for disqualification which included pregnancy week outside of target ranges, respondent was not pregnant, age outside study range (18–45), and others. Among those who were qualified, 99% agreed to participate, of those 77% completed the Baseline assessment, and 79% of those went on to complete at least 1 more assessment during the study.

**Table 3:** Sample disposition

Site intercept impressions	476,863		
Clicks on site intercept	5843		
Survey starts	5028	86.1%	% of clicks
Total disqualified^a ^	3471	69.0%	% of starts
Pregnancy week not within cohort targets	2186		
Did not complete the screening section	557		
Not pregnant	317		
Participating in other research	190		
Age outside range (< 18 or > 45)	151		
Ex-US	75		
Male	55		
Qualified	1557	31.0%	% of starts
Agree to participate	1535	98.6%	% of qualified
Completed baseline survey	1179	76.8%	% of agreed
Completed 1 + survey after baseline	934	79.2%	% of completed baseline

^a^Respondents could have > 1 disqualifier

There were some differences between women who completed 1 or 2 assessments and those who completed 3 or more. Those with fewer assessments tended to be younger, have slightly lower income, less education, and were more likely to be African American.

**Fig. 1:**
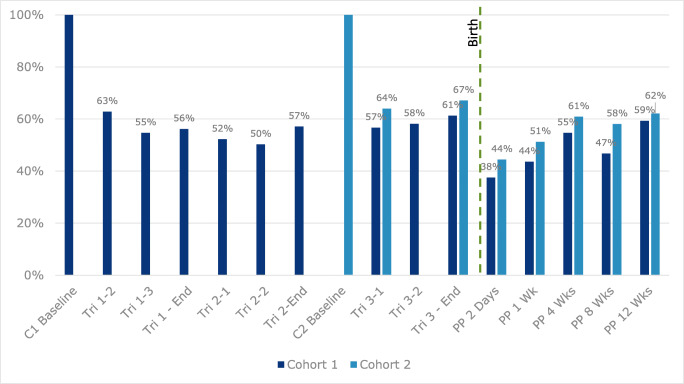
Completion rates by time period and cohort

Among the 858 women in cohort 1, 169 (19.7%) completed only the Baseline assessment; 80.3% completed 2 or more assessments; the majority of respondents (51%) completed 10 or more assessments; 157 (18.3%) completed all 15. In cohort 2, 76 women (23.6%) completed the Baseline only; 76.4% completed 2 or more assessments, and 1 in 3 (107, 33.2%) completed all 8. Among those with incomplete data, some women were lost to follow-up, others missed one or more assessments and returned to the study. Figure 1 shows the completion rate at each timepoint for both cohorts.

### Sample characteristics

The Baseline distributions of demographic characteristics are in Table 4. Study participants ranged in age from 18 to 44 with a median age of 30 years. More than 60% had a prior pregnancy. A larger number of women in cohort 2 reported being African American than did those in cohort 1 (26.4% vs. 17.1%; *p* < 0.001). The income distribution was similar across groups; the majority reported income of under $75,000. Nearly 80% of respondents were married or living with a partner. There were some differences with respect to employment; women in cohort 1 were more likely to be working full time (52.3% vs. 39.9%; p < 0.001), while those in cohort 2 were more likely to be unemployed (8.3% vs. 16.1%; *p* < 0.001). They were equally likely to report being stay-at-home moms (19.8% vs. 21.2%; *p* > 0.05). Educational attainment was similar across cohorts.

**Table 4:** Baseline demographics

		Cohort		
		1	2	Total
		(*n* = 858)	(*n* = 321)	*N* = 1179
		Column percent		
Age	18–24	22.7	18.3	21.5
	25–29	29.6	28.0	29.2
	30–34	29.3	33.5	30.4
	35–39	14.8	17.4	15.5
	40–44	3.6	2.8	3.4
Prior pregnancy		66.2	60.8	64.7
Have children		59.0	55.6	58.1
Ethnicity	African American	17.1	26.4	19.7
	Asia/Pacific Islander	4.9	8.7	5.9
	Caucasian	56.6	48.5	54.4
	Hispanic	18.4	14.3	17.3
Income	Under $25,000	23.2	26.5	24.1
	$25,000–$34,999	13.8	14.0	13.8
	$35,000–$49,999	10.8	10.6	10.8
	$50,000–$74,999	14.3	15.3	14.6
	$75,000–$99,999	11.4	10.9	11.3
	$100,000–$124,999	7.9	8.1	8.0
	$125,000 or higher	10.0	5.3	8.7
	Prefer not to answer	8.5	9.4	8.7
Marital status	Single	16.1	15.3	15.9
	Married	59.3	61.4	59.9
	Living with a partner	21.0	20.9	21.0
	Divorced	1.6	0.9	1.4
	Widowed	0.1	0.0	0.1
	Other	1.9	1.6	1.8
Employment	Full time	52.3	39.9	48.9
	Part time	12.3	15.2	13.0
	Unemployed	8.3	16.1	10.4
	Stay-at-home mom	19.8	21.2	20.2
	Student	5.5	4.4	5.2
	Other	1.9	3.2	2.2
Education	Some high school	4.1	5.0	4.3
	High school graduate	14.9	19.4	16.1
	Some college	32.8	33.9	33.1
	College graduate	29.4	21.6	27.3
	Some post-graduate	3.7	4.4	3.9
	Post-graduate degree	14.5	15.7	14.8

### Baseline physical and mental health and life experiences

The Baseline assessments included items about pregnancy experience, physical and mental health history, and life experiences during the prior year (Table 5). There were no differences in the reported mental health history or life experiences between the groups. More women in cohort 1 reported a history of obesity (11.9% vs. 7.5%; *p* < 001) and diabetes (either type) (2.3% vs. 0.3%; *p* < 0.01).

**Table 5:** Health history and life experiences

		Cohort 1	Cohort 2	
		Column percent		
Health Prior	Excellent/good	55.5	67.4	**^2^
	Fair/poor	11.9	6.8	
During this pregnancy	Fatigue or lack of energy	84.3	76.1	**
	Nausea	69.2	30.8	**
	Mood swings	48.5	46.9	
	Difficulty eating (e.g., motivation to eat healthy, appetite)	40.2	45.7	
	Back pain	38.2	59.0	**
	Headaches or migraines	38.2	34.8	
	Insomnia or trouble sleeping	35.9	57.8	**
	Anxiety	20.9	30.8	**
	Depressive symptoms	10.0	18.3	**
	High blood pressure	2.7	4.4	
	Difficulty managing your weight	0.0	17.4	**
	Gestational diabetes	0.0	9.0	**
Physical health history	Allergies	30.8	25.8	
	Migraines	16.3	16.5	
	Obesity	11.9	7.5	**
	Fertility problems	7.8	5.6	
	Sleep disorders	5.1	6.8	
	Hypertension	4.9	3.4	
	Irritable bowel syndrome (IBS)	4.1	5.0	
	Endometriosis	2.5	3.4	
	Diabetes (type I or II)	2.3	0.3	**
	Fibromyalgia	1.9	0.9	
Mental Health History	Substance addiction	0.4	0.9	
	Depressive symptoms	25.9	28.6	
	Generalized anxiety disorder	15.9	14.0	
	Panic attacks	14.8	14.3	
	Postpartum depressive symptoms	7.6	7.1	
	Post-traumatic stress disorder (PTSD)	7.5	5.9	
	Eating disorder	4.9	3.7	
	Bipolar disorder	4.1	5.6	
	Obsessive compulsive disorder (OCD)	3.4	2.5	
	Other emotional/mental health disorders	3.3	3.4	
Past year	Financial hardship	32.9	35.7	
	Death in family or of a close friend	29.0	27.6	
	Moved to a new location	26.6	31.1	
	Had a miscarriage	13.3	10.3	
	Personally lost a job	11.9	13.7	
	Divorced, separated or ended a relationship	9.7	8.7	
	Spouse/partner lost a job	8.3	13.4	**
	Legal problem/litigation	5.1	5.6	
	Victim of a non-violent crime (identity theft, burglary, etc.)	3.9	3.7	
	Victim of a violent crime	1.6	1.2	
	Victim of a natural disaster	1.0	1.0	

**^2^Top 2 and bottom 2 categories combined

**Significant *p* < 0.01

There were understandable differences between the cohorts in reported symptoms and diagnoses during the pregnancy, because cohort 1 responded early in the first trimester and cohort 2 in the third. Women in cohort 1 were more likely to report fatigue (84.3% vs. 76.1%) and nausea (69.2% vs. 30.8%). Those in cohort 2 were more likely to report: back pain (38.2% vs. 59.0%), insomnia (35.9% vs. 57.8%), anxiety (20.9% vs. 30.8%), depressive symptoms (10.0% vs. 18.3%), difficulty managing weight (0.0% vs. 17.4%), and gestational diabetes (0.0% vs. 9.0%), all *p* < 0.01.

### Depressive symptoms

#### Prevalent depressive symptoms

One in 4 women (27%) reported suffering from depression at any time prior to their pregnancy. Table 6 shows the categorical distribution of EPDS scores for each cohort at Baseline, at the end of each trimester, and the 4-week and 12-week postpartum assessments. At Baseline, almost 10% of the women in cohort 1 scored in the “Probable MDD” range. This increased to 13% at the end of the first and second trimesters and to almost 16% at the end of the third trimester. The proportion in this category returned to about 10% in both postpartum measures. For cohort 2, 12.7% at Baseline and 10.7% at end of the 3rd trimester met the criteria for “Probable MDD.” Following the birth of their children, the proportions in each cohort dropped to 9.6% and 8% respectively, not significantly different from each other. The pattern of a reduction following the birth of their children was similar in both groups.

**Table 6:** Cohort 1: EPDS distribution across pregnancy and postpartum periods

			Pregnancy				Postpartum	
			Baseline	T1	T2	T3	P4	E
Cohort 1	EPDS categories (% sample)	Unaffected	57.1	54.3	58.0	55.1	63.3	64.6
		Low prob. MDD	25.1	24.4	21.6	20.3	20.3	18.3
		Possible MDD	8.0	8.1	7.3	8.8	6.0	7.5
		Probable MDD	9.8	13.2	13.0	15.8	10.5	9.6
		Sample size (*n*)	858	484	491	526	469	509
			Baseline			T3	P4	E
Cohort 2	EPDS Categories (% sample)	Unaffected	46.9			51.4	63.1	66.0
		Low Prob. MDD	28.9			30.1	21.2	20.0
		Possible MDD	11.5			7.8	6.1	6.0
		Probable MDD	12.7			10.7	9.6	8.0
		Sample size (*n*)	322			216	198	200

T1–T4: [Core + OCI-R (set 4)] measurement end of trimester (T); P4: 4 weeks postpartum; E: end (final measurement)

#### Incident depressive symptoms

Incident depressive symptoms were defined as a score of 14 or higher on the EPDS (Probable MDD) after the Baseline assessment without a prior score in that range. All women with High Probability MDD at the Baseline assessment were considered prevalent cases. This is a conservative approach, necessary in the absence of other diagnostic information. We looked at incident depressive symptoms during pregnancy only in cohort 1 because the measurement period for cohort 2 during pregnancy did not include the first or second trimester. There were 176 incident cases in cohort 1.

**Fig. 2:**
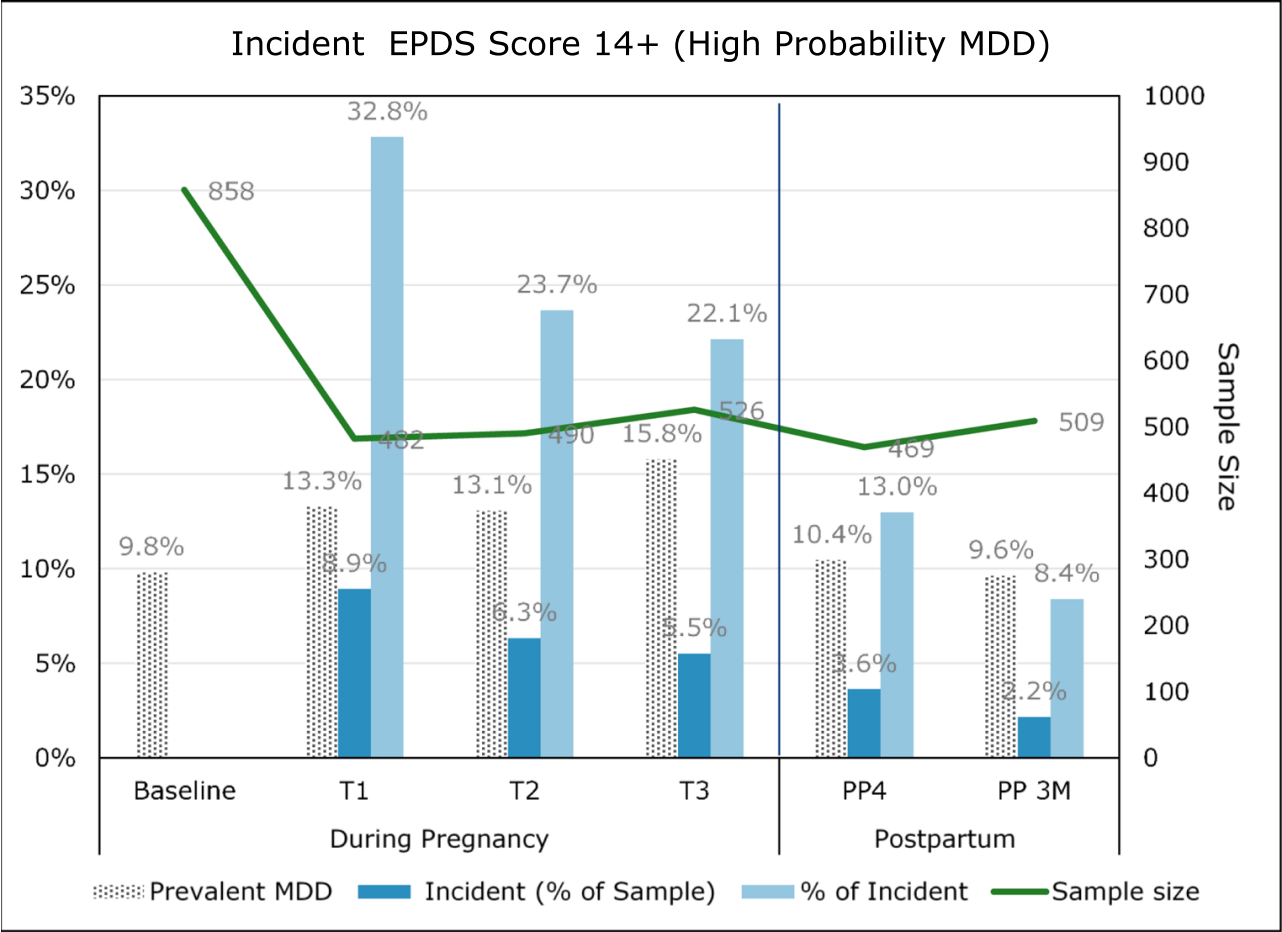
Cohort 1: incident depressive symptoms during pregnancy and postpartum periods

Figure 2 shows prevalent and incident depressive symptoms both as a proportion of the sample at each timepoint and as a proportion of all incident cases. Almost 80% of the incident cases occurred during pregnancy, 33% occurred in the first trimester, and 24% and 22% in the second and third trimesters, respectively. Women who scored in the High Probability MDD range at one timepoint did not necessarily persist at that level.

#### Predictors of incident depressive symptoms

A stepwise logistic regression predicting any onset of incident depressive symptoms showed that difficulty eating in the first trimester, a medical history including diagnoses of OCD and postpartum depressive symptoms, and moving or losing a job in the past year were associated with symptom onset (Table 7). The estimate for losing a job in the past year is protective with the upper end of the confidence limit close to 1 (0.98). Tests of the goodness-of-fit of the model were adequate (deviance and Pearson goodness-of-fit statistics *p* = 0.56, 0.37; Hosmer and Lemeshow goodness-of-fit test *p* = 0.69) indicating that the model fits the data.

**Table 7:** Logistic regression results predicting incident (new onset) MDD

Parameter	Estimate	Standard error	Wald chi-square	Pr > ChiSq	Odds ratio estimates		
					Estimate	95% conf limits	
Intercept	− 1.80	0.14	168.26	< 0.0001			
During: difficulty eating	0.54	0.17	9.61	0.00	1.71	1.22	2.41
Ever: OCD	0.95	0.40	5.49	0.02	2.58	1.17	5.71
Ever: PPD	0.87	0.29	9.14	0.00	2.38	1.36	4.17
Past year: moved	0.54	0.19	8.14	0.00	1.71	1.18	2.47
Past year: lost job	− 0.61	0.30	4.16	0.04	0.55	0.30	0.98

#### Kaplan–Meier survival estimates—time to incident depressive symptoms

**Fig. 3:**
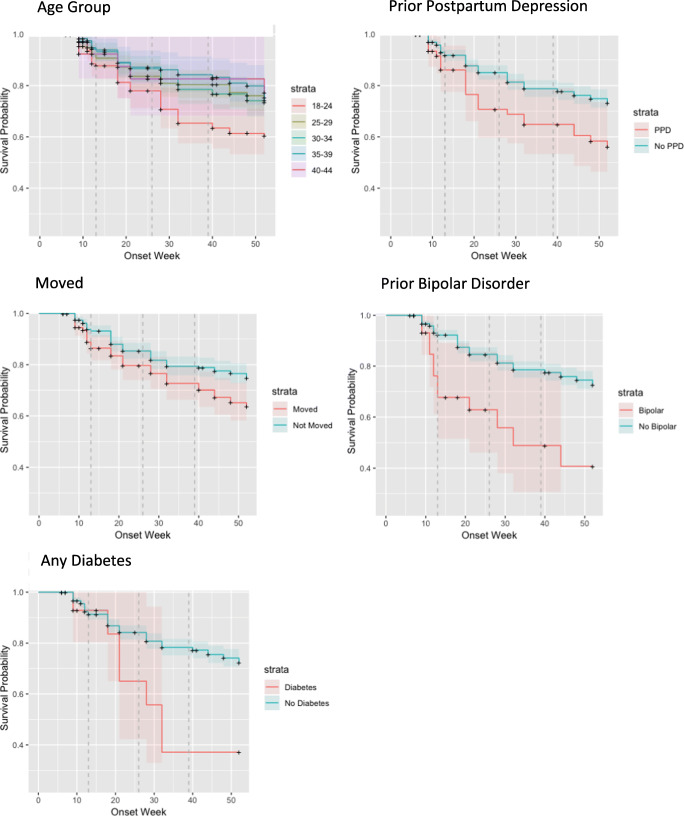
Time-to-onset curves for selected covariates

Survival graphs are presented in Fig. 3 which shows survival curves (time to incident symptoms) for age group, diabetes (any), prior postpartum depressive symptoms, prior bipolar disorder, and moved in the past year. A comparison of the age groups showed that the youngest group onset was significantly earlier than the oldest group (*p* = 0.03).

#### Predictors of time-to-onset—proportional hazards regression

The same set of independent variables was used to describe time-to-onset of incident symptoms. Predictors of time-to-onset in the multivariable model included history of bipolar disorder or postpartum depressive symptoms, moving in the past year, and difficulty eating and fatigue during the first trimester (Table 8). As was the case with the logistic regression, fatigue appeared to be protective.

**Table 8:** Proportional hazards regression predicting time-to-onset

Parameter	Parameter estimate	Standard error	Chi-square	Pr > ChiSq	Hazard ratio
Ever: bipolar disorder	0.74	0.31	5.68	0.02	2.11
Ever: PPD	0.57	0.23	6.10	0.01	1.77
Past year: moved	0.41	0.16	6.56	0.01	1.51
During: difficulty eating	0.41	0.16	6.94	0.01	1.51
During: fatigue	− 0.49	0.20	5.86	0.02	0.61

## Discussion

Closely following a cohort of pregnant women allowed us to observe incident symptoms during pregnancy and in the postpartum period. Contrary to commonly held beliefs, in this study, the large majority of incident symptoms occurred during pregnancy.

We assessed mood at up to 15 timepoints across pregnancy and the postpartum period and found that nearly 80% of incident depressive symptoms (EPDS score of 14 or higher) occurred during pregnancy. Fully 1/3 occurred in the first trimester. This pattern was also observed in the ALSPAC study in which fewer women had depressive symptoms in the postpartum than antenatal periods (Fergusson et al. 1996). The GUSTO study reported more depression in the postpartum period, but this may have been due to a lower cut-point on the EPDS after the birth (13 vs. 15 in the antenatal period) (Chong et al. 2014).

The Internet panel worked well; it gave us the ability to interview each woman at the same week of pregnancy with individually targeted assessments. The 2 cohorts were generally similar with respect to demographics, physical and mental health history, and past year experiences. They differed with respect to reports of mental and physical health complaints in the Baseline assessments. This is not unexpected because cohort 1 reports were made early in the pregnancy and cohort 2 near the end. It may be easier for pregnant women to participate in Internet-based assessments than to get out of the house for another pregnancy-related research appointment.

We employed an Internet-based panel to examine mental health during pregnancy through 3 months postpartum. Cohort 1 was ascertained during the first trimester and cohort 2 during their third trimester. The incentive system allowed us to retain more than half of the Baseline sample throughout the 15 measurements for cohort 1 and 8 for cohort 2. Furthermore, we were able to employ comprehensive psychometric assessments while respecting respondent burden.

Prevalence of MDD at the beginning of pregnancy was similar to that reported in the literature (1, 13–18). Prior medical history, pregnancy experience in the first trimester, and past year life experiences all contribute both to the likelihood of new onset depressive symptoms and to understanding when the onset will occur.

### Implications for intervention and treatment

Appreciating PND and time of onset is the new paradigm in maternal mental health; 80% of cases had antenatal onset in this work. Women should be screened with the first maternal health visit and monitored over the course of pregnancy and postpartum for signs of increased risk for PND. Proactive “generally regarded as safe” (GRAS) solutions need to be developed which can intercept the depressive symptoms before it can manifest.

If these findings are replicated, the mandate to find early interventions to prevent or delay the impact of depressive symptoms on both mothers and their offspring will be strengthened.

We believe that proactive screening for PND risk should initiate with the beginning of pregnancy and close monitoring should occur throughout the pregnancy and for several months post-childbirth. By identifying women at high risk for PND, it may be possible to provide safe and effective interventions to prevent, delay, or at least diminish the negative impact of the disease on both mother and child.

### Limitations

There are several limitations inherent in this work. First, the EPDS, while commonly used as a clinical assessment tool for mood in pregnancy, is not a validated diagnostic instrument. It will be important to replicate these findings using psychometric tools that are both valid in a pregnant population and have demonstrated utility for repeated measurement. Our implementation of the EPDS relied on self-report and did not involve a psychiatric evaluation by a clinician, nor did we have the advantage of any administrative claims or electronic health record information. Further, we used a shorter, 9-item version of the scale.

Major depressive episodes can have exogenous and endogenous etiologies with very different implications for diagnosis, course of illness, and treatment. We had insufficient data to distinguish the antecedents in women with incident symptoms.

Attrition was nontrivial, and 21% of the sample completed only the Baseline assessments; attrition did not appear to be completely at random, and women with higher EPDS scores were more likely to miss a subsequent assessment. As was the case with the EDEN study, attrition was higher among younger women, those with less education, and those who were unmarried. (Melchior et al. 2012).
